# Are the antagonist muscle fatigued during a prolonged isometric fatiguing elbow flexion at very low forces for young adults?

**DOI:** 10.3389/fphys.2022.956639

**Published:** 2022-10-07

**Authors:** Lejun Wang, Xiaoqian Song, Hua Yang, Ce Wang, Qineng Shao, Haifeng Tao, Minjie Qiao, Wenxin Niu, Xiaodong Liu

**Affiliations:** ^1^ Sport and Health Research Center, Physical Education Department, Tongji University, Shanghai, China; ^2^ School of Medicine, Tongji University, Shanghai, China; ^3^ School of Kinesiology, Shanghai University of Sport, Shanghai, China

**Keywords:** muscle fatigue, antagonistic muscles, prolonged isometric fatiguing contraction, muscle fiber conduction velocity, fractal dimension

## Abstract

The aim of this study was to examine whether antagonist muscles may be fatigued during a prolonged isometric fatiguing elbow flexion at very low forces. Twelve healthy male subjects sustained an isometric elbow flexion at 10% maximal voluntary contraction torque until exhaustion while multichannel electromyographic signals were collected from the biceps brachii (BB) and triceps brachii (TB). Muscle fiber conduction velocity (CV) and fractal dimension (FD) of both muscles were calculated to reflect peripheral and central fatigue. CV and FD of TB as well as FD of BB decreased progressively during the sustained fatiguing contraction, while the CV of BB declined at the beginning of the contraction and then increased progressively until the end of the contraction. The result may indicate that during the sustained low-force isometric fatiguing contraction, antagonist muscle may be peripherally fatigued, and changes in coactivation activities were modulated not only by central neuronal mechanisms of common drive but also by peripheral metabolic factors.

## Introduction

During human voluntary muscular contraction, the coactivation of antagonistic muscles concurrently happens with the contraction of agonistic muscles (De Luca and Mambrito, 1987;). The coactivation of antagonistic muscles is responsible for joint stiffness and contributes to joint stabilization and ligament protection (Wang et al., 2019). The coactivation level of antagonistic muscle can be influenced by various factors including muscle fatigue. In previous research studies, it has been interestingly observed that antagonist muscle coactivation activities may change in parallel with the changes of agonist muscle activity during submaximal isometric and isokinetic fatiguing contractions (Hunter et al., 2003; Duchateau and Baudry, 2014). The change of coactivation during muscle fatigue has been mainly attributed to the central adjustment mechanism of the common drive rather than fatigue-induced metabolic factors of the antagonist itself (Kotzamanidis, 2004).

Muscle fatigue can be defined as a reversible reduction in the neuromuscular system’s capacity to generate force or power (Vøllestad, 1997), which can arise from many points of the neuromuscular system and can be divided into central and peripheral fatigue according to its origin (McKenna and Hargreaves, 2008; Zając et al., 2015; Tornero-Aguilera et al., 2022). Surface electromyography (sEMG) has been widely used in muscle fatigue evaluation due to its non-invasiveness, real-time, and applicability (Karthick et al., 2018). In previous research studies, many indices based on sEMG analysis in time, frequency, and complexity domains have been suggested and used in muscle fatigue assessment (González-Izal et al., 2012; Dos Anjos et al., 2017; Rampichini et al., 2020). Among these indices, the enhancement of root mean square (RMS) amplitude may reflect the fatigue-related increase of motor unit recruit number, while the decline of median power frequency (MF) is related to the fatigue-induced decrease of motor unit firing rates. RMS and MF have been adopted as classical indicators to evaluate muscle fatigue. Moreover, by the research of Mesin et al. (2009), a bi-dimensional vector of muscle fiber conduction velocity (CV) and fractal dimension (FD) have been proposed to evaluate peripheral and central fatigue, respectively. The vector has been adopted by many research studies to assess muscle fatigue and may provide insights into the fatigue evaluation of antagonistic muscles.

According to the size principle as suggested by Henneman et al. (1965a; b), the coactivation of antagonist muscle may recruit mainly small size motor units as coactivation activities have been found to be lower level muscle activities. Therefore, antagonist muscles are apt to not fatigue during an isometric fatiguing contraction that lasted for a short time (Kotzamanidis, 2004), but when the sustained contraction time is prolonged, the results may be different. However, during prolonged fatiguing tasks, the fatigue of antagonist muscles has rarely been studied except for the research of Arellano et al. (2016), in which significant antagonist muscle fatigue was revealed for old adults rather than young adults after a fatiguing contraction of wrist flexors at 30% MVC sustained for 452 ± 174 s. As old adults have been suggested to have a modified control strategy of antagonist muscle coactivation compared to young adults, current literature struggles to provide a concrete conclusion on the fatigue of antagonistic muscles during a prolonged isometric fatiguing contraction at very low forces for young adults.

The aim of this study was to examine whether antagonist muscles may be fatigued during a prolonged isometric fatiguing elbow flexion at very low forces for young adults. RMS, MF, CV, and FD were observed during the sustained fatiguing contraction to reflect muscle fatigue development of BB and TB muscles. It was hypothesized that muscle fatigue of antagonist muscle may occur due to long-time coactivation activities, which can be reflected by changes of sEMG indices during prolonged isometric fatiguing contraction process.

## Materials and methods

### Subjects

Twelve right-handed male undergraduates (age: 21.1 ± 2.9 years; height: 177.3 ± 6.0 cm; weight: 64.5 ± 7.9 kg) volunteered to participate in this experiment. Participants were all healthy with no chronic pain or disease in relation to the neuromuscular system and were asked to avoid doing strenuous exercise the day before the experiment in order not to result in muscular fatigue or injury. Before the experiment, participants were fully informed of experimental procedures and potential risks during their involvement. The experiment was approved by the Ethics Committee of Tongji University (Ethics Committee approval number: 2020tjdx006).

### Data recording

#### Experimental setup

**FIGURE 1:**
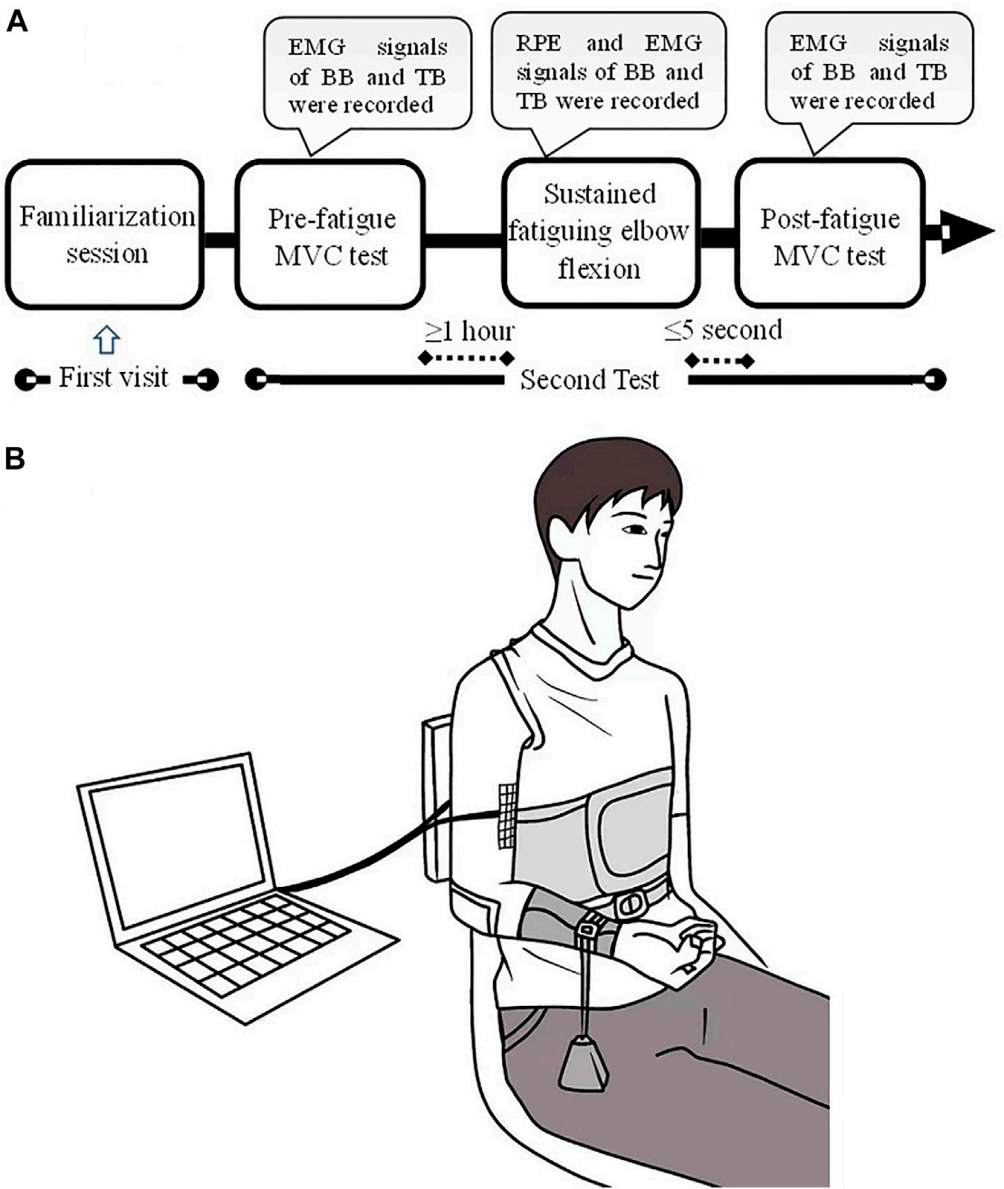
Experimental protocol and setup. A familiarization session was conducted for the first visit of the laboratory. The formal experiment comprised of three procedures: pre-fatigue MVC test, sustained fatiguing elbow flexion and post-fatigue MVC test **(A)**. During the experiment, the participants sat with the upper arm vertically placed, and the elbow angle was maintained at ∼90°. A weight was suspended from the distal part of the right forearm so as to produce a target of 10% MVC torque **(B)**.

A familiarization session was conducted for the first visit to the laboratory before the start of the test to introduce the experimental protocol and provide an opportunity for the subjects to try the test adopted in this study. The formal experiment comprised three procedures: first, participants performed an isometric maximal voluntary contraction (MVC) test of elbow flexion and extension with an isokinetic dynamometer (ConTrex AG, Dubendorf, Switzerland) to acquire the maximal isometric elbow flexion and extension torque of each subject without muscle fatigue. The MVC task involved increasing the elbow flexor/extensor torque gradually from zero to maximum over 3 s and maintaining the maximal value for 3 s before relaxing. The elbow flexor and extensor MVC test was performed alternately and was started in a randomized order to exclude the influence of order on the MVC torque. The elbow flexor/extensor MVC test was performed three times with a 5-min interval rest between two consecutive tests. Second, after a 15-min rest, each participant performed a sustained elbow flexion fatiguing contraction at 10% MVC torque of elbow flexion for as long as possible. Lastly, as soon as the fatiguing elbow flexion contraction task was completed, the subjects were instructed to perform elbow flexor and extensor MVCs. The protocol of the post-fatigue test was the same as that in the pre-fatigue test. All motor tasks were finished with the right limb. The experimental protocol and setup are depicted in Figure 1.

During the experiment, the participants sat with their upper arms vertically placed, and the elbow angle was maintained at ∼90°. The trunk was fixed by bandages, while the right forearm was positioned parallel to the ground and supinated. During the sustained elbow flexion fatiguing contraction, a weight was suspended from the distal part of the right forearm so as to produce a target of 10% MVC torque. The participants were instructed to maintain their angle of the elbow joint as close as possible at ∼90° until they experienced exhaustion and were no longer able to hold the posture. The participants were informed to adjust the elbow angle once the angle was greater than 100° during the task. Elbow angle was monitored by a camera, and the task failure was determined based on the criterion of *1*) elbow angle ≥110° and *2*) the adjust time of elbow angle ≥10 s. The participants were verbally and vigorously encouraged to continue the sustained contraction as long as possible. During the sustained contraction, multichannel sEMG signals of the biceps brachii (BB) and triceps brachii (TB) muscles were recorded. Moreover, the rating of perceived exertion (RPE) of each subject was recorded for every 2-min duration time during the sustained elbow flexion task using the Borg’s CR-10 scale.

#### EMG measurement

Multichannel EMG signals were recorded from the BB and TB muscles of the right arm in a single differential configuration using adhesive electrode arrays (64 electrodes, 8-mm IED, OT Bioelettronica, Torino, Italy). Before the placement of the electrodes, the skin was shaved and cleaned with alcohol in accordance with the SENIAM’s recommendation for skin preparation. The optimal position and orientation of the array were sought for each muscle on the basis of visual inspection of the sEMG signals (Marco Barbero, 2012). The adhesive electrode arrays were placed parallel to muscle fibers. To assure proper electrode–skin contact, electrode cavities of the arrays were filled with conductive gel. The electrode arrays were fixed using extensible dressing. The sEMG signals were detected in a monopolar mode, with a sampling rate of 2,048 Hz, an A/D resolution of 16 bits, and a bandpass filter of 10–500 Hz.

### Data processing and analysis

Multichannel signals recorded from both BB and TB muscles were visually inspected in order to reject channels with obvious artifacts. RMS and MF were calculated for non-overlapping epochs of 2.048 s duration time for each channel sEMG during the fatiguing contraction according to previous research (Wang et al., 2015).

CV was computed off-line with numerical algorithms (Merletti et al., 1990) using non-overlapping signal epochs of 0.5 s. CVs were calculated among all the accepted channels and were calculated as *i/d*, where *i* is the interelectrode distance and *d* is the delay time between the signals obtained from the two adjacent electrodes. The value of d was determined based on the time shift required to minimize the mean square error between the Fourier transforms of the two-channel EMG signals. The correlation coefficient between sEMG signals recorded from two adjacent electrodes was calculated. Referring to previous research (Boccia et al., 2015), recorded signals were excluded from the analysis if the correlation coefficient between the two adjacent double differential signals was less than 0.80.

FD was computed on each of the signals selected for CV computation and then averaged. FD was calculated using the box-counting method referenced in previous research studies (Gitter and Czerniecki, 1995; Mesin et al., 2009). A grid of square boxes was used to cover the signal, and the number of boxes that the sEMG waveform passes through was determined. The range of box size was restricted in order to avoid saturation for both high and low values of size (Gitter and Czerniecki, 1995). The box size was fixed to 13 steps equally spaced on a logarithmic scale, with the smallest box equal to 1/128th of a second and the largest box equal to 1/8th of a second (Beretta-Piccoli et al., 2017). The vertical side of the boxes was normalized to the range of the signal during epochs of 1 s and divided into the same number of boxes.

FD is defined as the following formula:

$$FD=\frac{\log N}{\log\frac{1}{L}},$$

where N is the number of boxes required to cover the signal and L is the box side, with the ratio indicating the slope of the interpolation line.

After the calculation of sEMG indices in each epoch, the total number of epochs was equally divided into 10 contraction periods, and values in the same period were averaged. Based on the previous averaging, the results of RMS, MF, CV, and FD were time based normalized to 100% duration time for each subject. Results of RMS were normalized to the maximal value of the MVC test, while MF, CV, and FD were normalized relative to the maximum and minimum values of the contraction, averaged among all the accepted channels. Data were analyzed by custom-written software in MATLAB R2016a (MathWorks, Natick, MA, United States).

### Statistical analysis

SPSS 19.0 for Windows was used for statistical analysis (IBM, United States). The normality test was conducted by the Kolmogorov–Smirnov test. Mauchly’s test of sphericity was used to test whether or not the assumption of sphericity is met in a repeated measure ANOVA. MVC torque, RPE, RMS, MF, CV, and FD were all tested to follow a normal distribution and the assumption of sphericity (*p* > 0.05). Therefore, a repeated-measures analysis of variance [within factors: contraction duration time] was used to determine the significance of RPE, RMS, MF, CV, and FD during fatiguing contraction. The significance of MVC torque was examined by a two-factor repeated-measures analysis of variance [within factors: contraction directions (elbow flexion vs. extension) and fatigue (pre- vs. post-fatigue)]. Pearson’s cross-correlation analysis was used to determine the correlation between all indicators and contraction duration time. Statistical significance was set to *α* = 0.05. Data were presented as mean ± SD.

## Results

### MVC torque of elbow flexion and extension before and after fatiguing contraction

**FIGURE 2:**
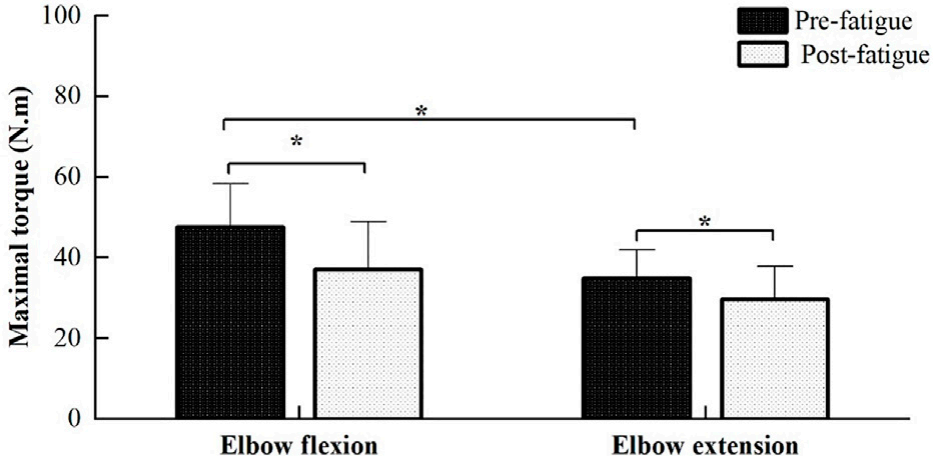
Comparisons of MVC torques of elbow flexion and extension between the pre- and post-fatigue tests. The MVC torques of elbow flexion were significantly higher than those of elbow extension for the pre-fatigue test (*p* ≤ 0.001), while MVC torques in the post-fatigue test were found to be significantly lower than those of the pre-fatigue test for both elbow flexion and extension (*p* ≤ 0.001).

The average duration time of fatiguing contraction is 1,177 ± 365 s, ranging from 642 to 1,810 s. Figure 2 illustrated the comparisons of MVC torques of elbow flexion and extension between the pre- and post-fatigue tests. MVC torques of elbow flexion during the pre- and post-fatigue tests were 47.61 ± 10.76 and 37.17 ± 11.68 N.m, and 34.88 ± 7.02 and 29.70 ± 8.13 N.m for elbow extension contraction. Significant main effects of both contraction directions (elbow flexion vs. extension) and fatigue (pre- vs. post-fatigue) on MVC torques were observed (contraction directions: F = 54.662, *p* ≤ 0.001; fatigue: F = 67.122, *p* ≤ 0.001). Moreover, a significant contraction direction (elbow flexion vs. extension) by fatigue (pre- vs. post-fatigue) interaction effect was found (F = 6.322, *p* = 0.026). The post-comparison results showed that the MVC torques of the elbow flexion were significantly higher than those of elbow extension for the pre-fatigue test, while no significant difference in MVC torque was observed between elbow flexion and elbow extension for post-fatigue (pre-fatigue test: *p* ≤ 0.001, post-fatigue test: *p* = 0.061), and MVC torque of both elbow flexion and extension significantly decreased during the post-fatigue test than that of the pre-fatigue test (elbow flexion: *p* ≤ 0.001, elbow extension: *p* ≤ 0.001).

### Rating of perceived exertion, EMG RMS, and MF changes during the fatiguing elbow flexion contraction

**FIGURE 3:**
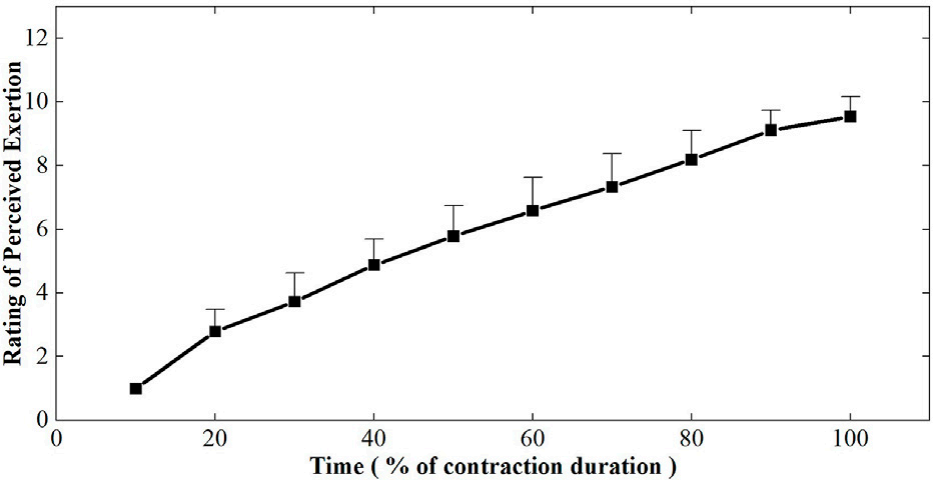
Changes of rating of perceived exertion plotted as a percentage of contraction time during the sustained fatiguing contraction. For each subject, data have been normalized relative to the initial values.

Figure 3 depicted the changes in rating of perceived exertion plotted as a percentage of contraction time during the sustained fatiguing contraction. RPE increased progressively during the whole process of fatiguing contraction and reached 9.54 ± 0.63 at the last 10% contraction duration time. A repeated-measures analysis of variance revealed that RPE had a significant difference in 10 contraction periods (F = 258.764, *p* ≤ 0.001). A significant increasing tendency of the RPE with the change in contraction duration time was found by Pearson cross-correlation analysis (*r* = 0.946, *p* ≤ 0.001).

**FIGURE 4:**
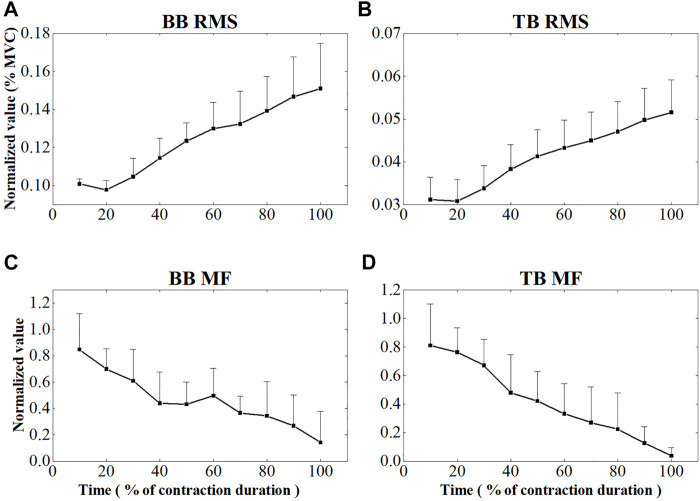
EMG RMS and MF changes of BB **(A,C)** and TB **(B,D)** muscle during the sustained fatiguing contraction. For each subject, data of EMG RMS and MF has been normalized relative to the maximum and minimum values of the contraction and the time base has been normalized to 100 %.

Figure 4 showed the EMG RMS and MF changes of BB and TB muscles during the sustained fatiguing contraction. EMG RMS increased and the EMG MF decreased progressively during the sustained fatiguing contraction for both BB and TB muscles. Repeated-measures analysis of variance revealed that EMG RMS of both BB and TB muscles had a significant difference in 10 contraction periods (BB: F = 84.132, *p* ≤ 0.001; TB: F = 122.248; *p* ≤ 0.001), as well as EMG MF of both BB and TB muscles (BB: F = 10.916, *p* ≤ 0.001; TB: F = 18.990; *p* ≤ 0.001). In addition, a significant positive correlation between EMG RMS and contraction duration time and a significant negative correlation between EMG MF and contraction duration time were observed in both BB and TB muscles (BB-RMS: *r* = 0.908, *p* ≤ 0.001; TB-RMS: *r* = 0.936, *p* ≤ 0.001; BB-MF: *r* = −0.639, *p* ≤ 0.001; TB-MF: r = −0.758, *p* ≤ 0.001).

### CV and FD changes during the fatiguing elbow flexion contraction

**FIGURE 5:**
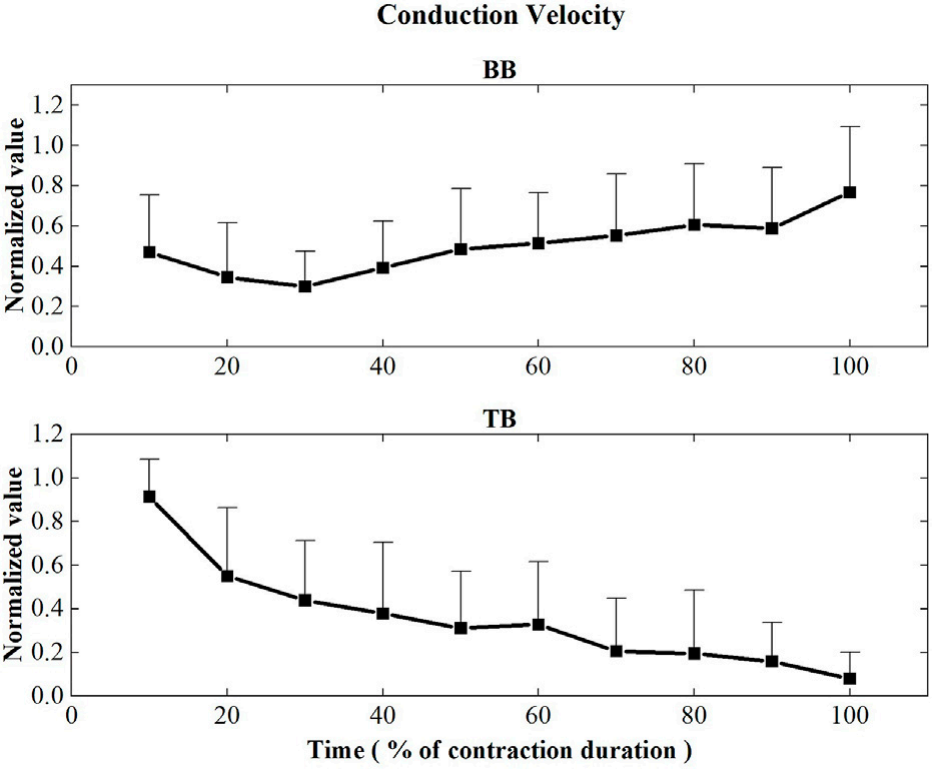
Conduction velocity values of the BB and TB muscles during the sustained fatiguing contraction. For each subject, data have been normalized relative to the maximum and minimum values of the contraction, and the time base has been normalized to 100%.

The initial values of muscle fiber CV and FD during the sustained fatiguing contraction were showed in Table 1. Figure 5 represents the CV values of BB and TB muscles during the sustained fatiguing contraction. For BB muscle, CV showed a significant decreased tendency in the first three contraction periods (*r* = −0.347, *p* = 0.048), while it significantly increased for the rest of contraction duration (*r* = 0.407, *p* ≤ 0.001). For TB muscle, CV decreased progressively during the whole sustained fatiguing contraction (*r* = −0.585, *p* ≤ 0.001). Repeated-measures analysis of variance illustrated that significant differences were revealed for the CV of BB among the first three contraction periods and the rest of the contraction duration, as well as the CV of TB among the whole contraction duration time.

**TABLE 1:** Initial values of muscle fiber CV and FD during the sustained fatiguing contraction.

	BB	TB
CV (m/s)	5.73 ± 0.15	3.18 ± 0.12
FD	1.69 ± 0.04	1.72 ± 0.03

**FIGURE 6:**
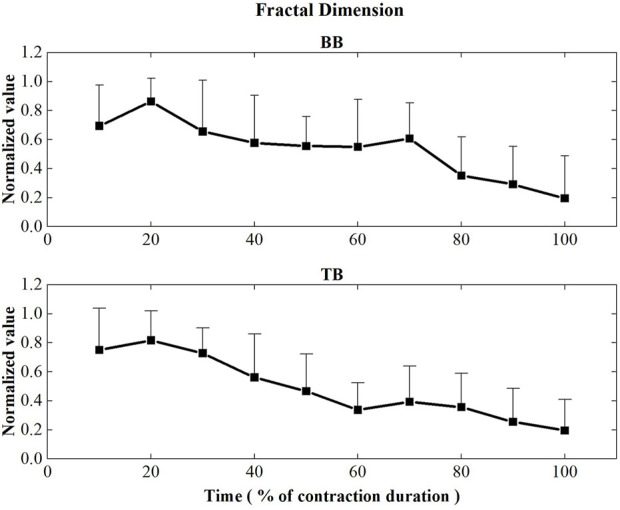
Fractal dimension values of the BB and TB muscles during the sustained fatiguing contraction. For each subject, data have been normalized relative to the maximum and minimum values of the contraction, and the time base has been normalized to 100%.

Figure 6 displayed the FD values of BB and TB muscles during the sustained fatiguing contraction. For both BB and TB muscle, FD showed significant decreased tendency with the increase of contraction duration time (BB: *r* = −0.512, *p* ≤ 0.001; TB: *r* = −0.633, *p* ≤ 0.001). Repeated-measures analysis of variance revealed that a significant main effect of the contraction period factor on FD of both BB and TB muscles was observed (BB: F = 6.434, *p* ≤ 0.001, TB: F = 9.610, *p* ≤ 0.001).

## Discussion

The current study was designed to examine whether antagonist muscle may fatigue during a prolonged isometric fatiguing contraction. It was interesting to find that the CV and FD of antagonist muscle decreased progressively during the sustained fatiguing contraction, while the maximum joint torque of antagonist muscle significantly decreased after sustained muscle fatigue contraction. The results may demonstrate that during the prolonged fatiguing contraction, antagonist muscles do fatigue as a result of coactivation. As far as we know, it is the first time to reveal the fatigue of antagonist muscle during a prolonged fatiguing contraction at low forces.

During the sustained fatiguing elbow flexion contraction, the EMG RMS of the BB muscle increased and the EMG MF decreased progressively. The increase of EMG RMS may indicate increases in motor unit recruit number to compensate for the decline of muscle contractility induced by fatigue, while the decline of MF is mainly related to the changes in both the motor unit firing rate and the muscle fiber CV induced by muscle fatigue (Cifrek et al., 2009; Enoka et al., 2011). These changes are typical characteristics of agonistic muscle EMG signals during fatiguing submaximal isometric contractions (Gandevia, 2001; Lacerda et al., 2019; Miller et al., 2020). Meanwhile, EMG RMS and MF of antagonistic muscle TB showed the same changing tendency when compared to BB. All these results are consistent with previous research studies (Ebenbichler et al., 1998; Mullany et al., 2002; Levenez et al., 2005; Levenez et al., 2008; Troiano et al., 2008). However, in previous research studies, the abovementioned changes in TB muscles have been attributed to the central adjustment mechanism of common drive rather than the fatigue-induced metabolic factors of the antagonist itself (Booghs et al., 2012).

In this study, the FD of both BB and TB muscles showed a significant decrease tendency with the sustained contraction time. It has been revealed that FD was the EMG parameter, which is most related to the level of synchronization, so it is a promising index of central response to fatigue (Troiano et al., 2008; Mesin et al., 2009; Boccia et al., 2015). However, as suggested by Rampichini et al. (2020), the interference nature of the EMG signal makes the speculation on the origin of central fatigue components questionable (Rampichini et al., 2020). Nevertheless, the results found in the current study may give insights into the understanding of central fatigue development related to BB contraction and TB coactivation. It has been revealed that sustained isometric fatiguing contraction at low forces produces prominent central fatigue, thus the central fatigue of BB muscle in this study can be easily expected (Smith et al., 2007). However, when related to antagonist muscles, a control strategy of shared neural input between agonist and antagonist muscles, which is referred to as common drive, has been suggested since the research of De Luca (De Luca and Mambrito, 1987; De Luca and Erim, 2002). According to the theory, the changes in EMG activities for antagonist muscles may be partly explained by central common drive modulation.

In previous research, a strict relation between the changes of CV and MF during sustained fatiguing contraction has been revealed (Lowery et al., 2002). In the current research, EMG MF of both BB and TB muscles decreased progressively during the sustained fatiguing contraction. However, the CV of BB and TB showed different changing trends during the sustained contraction. As the decline of MF has been suggested to be mainly related to the changes in both the motor unit firing rate and the muscle fiber CV induced by muscle fatigue (Cifrek et al., 2009; Enoka et al., 2011), the different changing trends of CV may be related to the difference of motor unit firing rates between BB and TB muscles during the sustained contraction.

During the prolonged fatiguing contraction, the CV of BB muscle declined during the initial 30% of contraction duration and then increased progressively until the end of the contraction, while the CV of TB muscle showed a continuous decrease tendency for the whole contraction. During sustained isometric contractions, CV has been found to decrease due to factors including an increase in muscle acidosis (Myburgh, 2004; Schmitz et al., 2012), alterations in blood flow (Sj Gaard et al., 1988; Hammer et al., 2020), a decrease in extracellular sodium concentration (Overgaard et al., 1997), and accumulation of extracellular potassium ions (Fortune and Lowery, 2009; Whitten et al., 2021) as a result of peripheral muscle fatigue. On the other hand, CV increases gradually when larger motor units are recruited (Adam and De Luca, 2003). According to the size principle, small motor units should be recruited for both BB and TB muscles at the initial stage of sustained contraction in this study. The decline of CV at the beginning of the contraction for BB muscle can be explained by the abovementioned peripheral factors of performance fatigability, while the latter increase of CV may be related to the new recruitment of fresh, higher threshold unfatigued motor units to compensate for muscle fatigue and maintain the motor task.

It has been revealed that the percentage of type II fibers in the triceps brachii was 64.6 ± 3.2%, while the percentage of type II fiber area was 71.4 ± 2.7% (Terzis et al., 2003). However, the coactivation level of TB (relative to the EMG activation level of the MVC test) may be less than 10% of the MVC contraction level during the sustained elbow flexion contraction. Therefore, slow twitch muscle fibers (type I fiber) would be mainly recruited for the coactivation of TB during the whole sustained contraction. The decrease in CV during the sustained contraction may indicate a steady development of peripheral fatigue and metabolic changes for TB muscle as a result of coactivation. While the common drive control strategy of coactivation has been mainly accepted, different viewpoints have been suggested in previous research studies. Specifically, it has been indicated that the level of coactivation during a fatiguing contraction of elbow flexion is mediated by supraspinal rather than spinal mechanisms and involves differential control of agonist and antagonist muscles. The peripheral fatigue of antagonist muscle as a result of coactivation seemed to provide an explanation for the existence of other control paths of coactivation except for the common drive strategy.

However, the absence of electrical/magnetic stimulation research methods to detect muscle fatigue in central and peripheral components should be acknowledged. Electrical/magnetic stimulation has been accepted to be the only validated method to detect central and peripheral components of fatigue, while the interference nature of the EMG signal may make the detection of central and peripheral fatigue only by the EMG method questionable (Rampichini et al., 2020). Nevertheless, the current research has revealed the performance fatigability of antagonist muscles as a result of prolonged isometric fatiguing elbow flexion at very low forces for young adults, and the speculation of central and peripheral fatigue by EMG signals in the current study may at least give insights into the understanding of central and peripheral fatigue development during the sustained contractions.

In conclusion, antagonist muscles may be peripherally fatigued during the sustained isometric fatiguing contraction at low forces. The result may indicate that changes in coactivation activities were modulated not only by central neuronal mechanisms of common drive but also by peripheral metabolic factors.

## Data Availability

The raw data supporting the conclusions of this article will be made available by the authors, without undue reservation.
